# Supplementary material for: Glycoprofiling of proteins as prostate cancer biomarkers: A multinational population study
Source: PLoS One. 2024 Mar 18;19(3):e0300430. doi: 10.1371/journal.pone.0300430 (PMC10947713; doi:10.1371/journal.pone.0300430)
Supplement: S1 File — The file contains the following captions: Preparation of glycoprotein standard (GPS); Standardised glycopsy immunoassay (GIA) test; Validation of the streptavidin antibody; Analyses and biostatistics; Glycoprofiling of ZA2G; the following figures: S1 Fig: Schematic presentation of the GIA assay; S2 Fig: Evaluation of glycoprotein standard (GPS); S3 Fig: Calibration curve for fPSA_tPSA and GIA for trains and test subsets; and the following Tables: S1 Table: Summary of coefficients, standard errors, z score and significance probabilities p for each marker in the GIA test.; S2 Table: Significance of lectins recognizing gPSA4 and gPSA2 in the final GIA model compared to tPSA+fPSA only using Wald test. (DOCX) [file pone.0300430.s001.docx]

**SUPPORTING INFORMATION**

**Glycoprofiling of proteins as prostate cancer biomarkers: a multinational population study**

Andrea Pinkeova^1^, Adela Tomikova^1^, Aniko Bertokova^1^, Eva Fabinyova^1^, Radka Bartova^1^, Eduard Jane^1,2^, Stefania Hroncekova^2^, Karl-Dietrich Sievert^3^, Roman Sokol^4^, Michal Jirasko^5^, Radek Kucera^5,6^, Iris E. Eder^7^, Wolfgang Horninger^7^, Helmut Klocker^7^, Petra Ďubjaková^8^, Juraj Fillo^8^, Tomas Bertok^1,2^, Jan Tkac^1,2^*

^1^Glycanostics, Ltd., Kudlakova 7, Bratislava 841 01, Slovak Republic;

^2^ Institute of Chemistry, Dubravska cesta 9, Bratislava 845 38, Slovak Republic;

^3^ Klinikum Lippe - Clinic for Urology, Roentgenstraße 18, Detmold 32756, Germany;

^4^ Private Urological Ambulance, Piaristicka 6, Trencin 911 01, Slovak Republic;

^5^ Department of Pharmacology and Toxicology, Faculty of Medicine in Pilsen, Charles University, Pilsen 323 00, Czech Republic.

^6^ Department of Immunochemistry Diagnostics, University Hospital in Pilsen, E. Benese 13, Pilsen 301 00, Czech Republic;

^7^ Department of Urology, Division of Experimental Urology, Medical University Innsbruck, Anichstrasse 35, A-6020 Innsbruck, Austria;

^8^ University Hospital Bratislava, Mickiewiczova 13, 811 07 Bratislava, Slovakia;

* Corresponding author: [jan.tkac@glycanostics.com](mailto:jan.tkac@glycanostics.com); [jan.tkac@savba.sk](mailto:jan.tkac@savba.sk) (JT)

**Preparation of glycoprotein standard (GPS)**

Biotinylated glycan (LacdiNAc-C3-biot abbreviated as *LDN*; GlycoNZ, New Zealand), KH_2_PO_4_ and K_2_HPO_4_ solutions (from Sigma Merck, USA), streptavidin (Vector Labs, USA) and Zeba™ Spin Desalting Columns (MWCO 7k, Thermo, USA) were used for the preparation of the standard for calibration of the GIA test, GPS, consisting of streptavidin to which biotinylated glycan molecules have been bound.

1 mg of a lyophilised streptavidin powder was re-suspended in 1 mL of sterile/filtered 0.1 M PB (phosphate buffer) pH 7.4 to obtain a concentration of 1 mg mL^-1^. Glycan-conjugated streptavidin was prepared by mixing 100 µL of streptavidin solution with 100 µL of a biotinylated glycan solution (50 µL of a biotinylated glycan stock solution, diluted by 480 µL of sterile PB buffer). The mixture was vortexed for 5s and then incubated using a gentle mixing (500 rpm) for 1h at room temperature (25 °C). The end result was streptavidin conjugated with 4 molecules of biotinylated glycans. The method was used to prepare the GPS in which the localisation of glycans on the streptavidin protein backbone was precisely controlled, as were the number of glycan epitopes on the protein backbone.

After the conjugation process, an excess of the glycan was removed from the final solution using size-exclusion chromatography and the final concentration of GPS was determined by measuring the absorbance at 280 nm (A_280_) and comparing it with the theoretical value. A GPS solution (the streptavidin/[LDN]_4_) with c = 10 ng mL^-1^) was prepared prior to any measurement and the signal of each sample was divided (normalised) by the signal intensity of GPS when measured on the same plate. Each sample was measured in triplicates with a final RSD not exceeding 10%.

**Standardised glycoprotein immune (GIA) test**

The GIA test is based on the application of an antibody against the protein of which glycosylation is analysed (a-P, i.e*.,* anti-fPSA or anti-ZA2G linked to horse radish peroxidise modified magnetic beads (HRP-MBs) (**Fig. 1 upper row,** *a-P*). When HRP-MB-anti-fPSA beads are incubated with a serum sample, fPSA is attached to the MBs through anti-fPSA. In the subsequent step, fPSA attached to the modified MBs is incubated with lectin modified ELISA plate to make a sandwich in which fPSA is sandwiched between lectin and anti-fPSA (present on HRB_MBs). HRP is then used for a signal generation

The GPS was designed to function in a similar way to fPSA on an ELISA plate: it is sandwiched between the lectin and an anti-streptavidin antibody (a-GPS, **Fig. S1 lower row**). Since streptavidin was applied as a protein scaffold for the glycan standard, it was essential to identify anti-streptavidin antibodies (abbreviated in **Fig. S1 lower row** as *a-GPS*) that bind the streptavidin occupied by 4 molecules of glycans. This will attach the standard to the magnetic beads to generate a sandwich configuration.

The GPS was included in each GIA test and used for the normalisation of the signal obtained from serum samples: i.e. ${signal}_{NORM}=\frac{\bar{A}}{B}$ with ($\bar{A}$) = sample signal and ($\bar{B}$)= GPS signal (**Fig. S1**). A short-term stability test revealed that the GPS was stable only when stored at 4°C, but not when stored in a lyophilised state at -20°C (data not shown). The reproducibility of the GPS preparation was excellent with RSD (%) of ~8.8%. Therefore, the GPS could be applied for the calibration of the GIA test, when stored in a concentrated form at 4°C for 5 days during clinical validation studies.


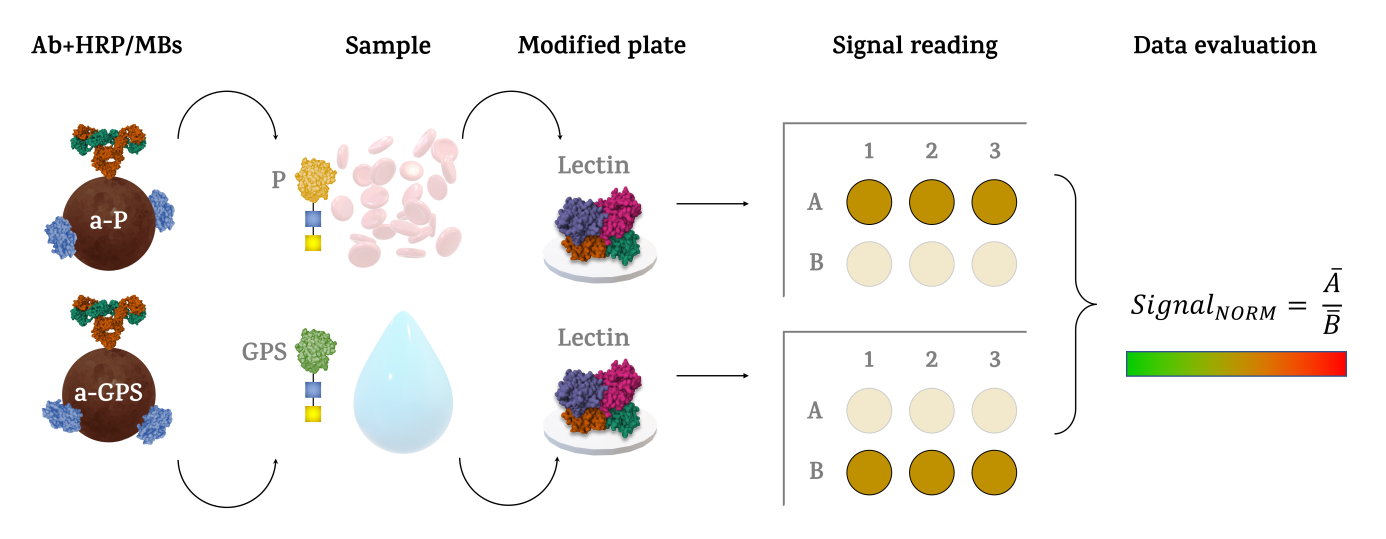


**S1 Figure:** Schematic presentation of the GIA assay. In the first step, antibody against fPSA protein (a-P) and peroxidase (HRP depicted in blue) attached to magnetic beads (MBs) are added to the sample containing fPSA. MB with captured fPSA are introduced into a lectin modified ELISA plate to create a sandwich with generation of a signal ($\bar{A}$). In parallel, antibody against glycoprotein standard (a-GPS) attached to HPR-MBs are incubated with GPS. MBs with captured GPS are introduced into a lectin modified ELISA plate to create a sandwich that generates a signal ($\bar{B}$) to normalise the signal from the measurements of the samples.

**Validation of the streptavidin antibody**

To control proper function of the streptavidin antibody a Surface Plasmon Resonance (SPR) experiment (Biacore X100, CM5 SPR chip and all reagent supplemented by GE Healthcare) was performed as described in **Fig. S2**. A CM5 chip was modified with a lectin (WFL) using a common EDC/NHS amine coupling), followed by blocking with ethanolamine. Subsequently GPS was injected into a measuring flow cell (control cell remained empty). Finally, the sandwich was completed with an injection of anti-streptavidin antibody. The signal difference after dissociation phase was ~1700 RU between the two cells, suggesting a strong binding of anti-streptavidin to the GPS in a sandwich configuration.


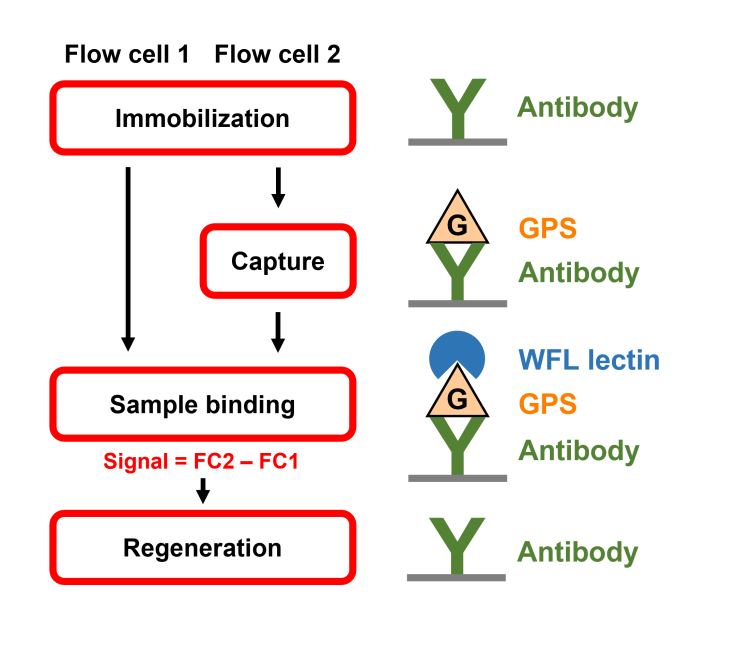

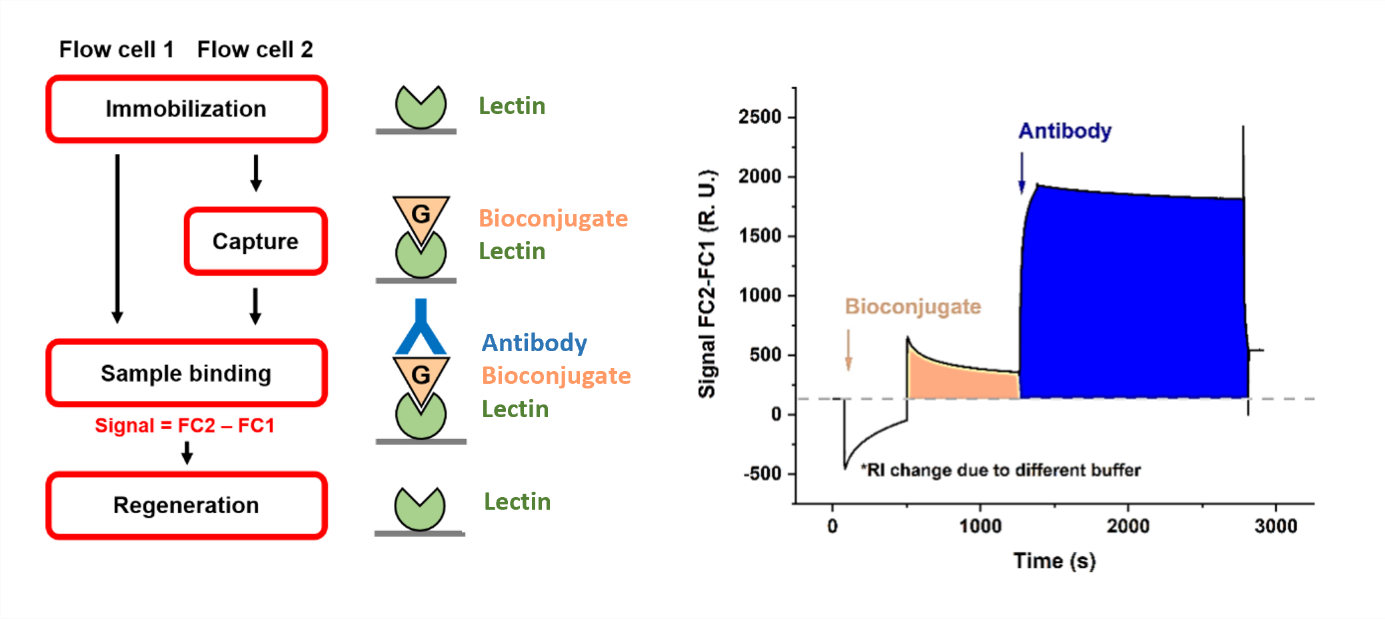


**S2 Figure:** Evaluation of glycoprotein standard (GPS). **Left part of the image:** Lectin WFL was immobilised into two flow cells. GPS was injected into flow cell 2 where it was bound to WFL lectin. Flow cell 1 remained occupied by the lectin only. Then, anti-streptavidin antibody was injected into both flow cells and the interface was finally regenerated. **Right part of the image:** SPR sensorgram with injection of GPS to WFL modified SPR chip with response highlighted in orange. Then, anti-streptavidin antibody was injected into both flow cells with strong binding response highlighted in blue.

**Analyses and biostatistics**

The serum levels of total PSA (tPSA)and free PSA (fPSA) and fPSA% (measured by highly automatic assay systems provided by Beckman Coulter, Roche and Siemens) were provided by the 4 recruiting clinical centres together with the clinical data. Subsequently, fPSA% and the PHI were calculated using the formulas: fPSA% = (fPSA/tPSA) × 100 and PHI = (−2proPSA/fPSA) × √tPSA. fPSA% was calculated using the formula fPSA%=(fPSA/tPSA)*100.

GIA ROC curve were computed by integrating fPSA glycoprofiles analysed with the two lectins (fPSA^WFL^; fPSA^PHA-E^) together with tPSA and fPSA [20]. Decision curves were computed using a dcurves package ^1^. All confidence intervals (CIs) presented are 95% two-sided bootstrap intervals.

**S1 Table:** Summary of coefficients, standard errors, z score and significance probabilities p for each marker in the GIA test.

|  | **Coefficients** | **SE** | **z-value** | **p-value** |
| --- | --- | --- | --- | --- |
| **(Intercept)** | -2.836 | 0.904 | -3.138 | 0.002 |
| **PSA** | 0.677 | 0.081 | 8.408 | 0 |
| **fPSA** | -2.451 | 0.402 | -6.091 | 1.124e-09 |
| fPSA^WFL^ | 2.665 | 0.601 | 4.437 | 9.118e-06 |
| fPSA^PHA-E^ | -2.402 | 0.839 | -2.862 | 0.004 |

**S2 Table**: Significance of lectins recognizing gPSA4 and gPSA2 in the final GIA model compared to tPSA+fPSA only using Wald test. Df = degrees of freedom, ChiSq = chi squared

| **Model** | **Df** | **ChiSq** | **p-value** |
| --- | --- | --- | --- |
| PSA+fPSA | NA | NA | NA |
| PSA+fPSA+ fPSA^WFL^+fPSA^PHA-E^ | 2 | 26.739 | 1.562e-06 |

The calibration curves for PSA+fPSA and PSA+fPSA+ fPSA^WFL^+fPSA^PHA-E^ models follow the diagonal line (y = x), indicating good alignment between predicted and observed values. However, in higher values of the mean predicted probability, calibration curves deviate slightly from the diagonal line due to a slight imbalance in the compared groups (the PCA sub-group is smaller). None of the curves show an S shape or bias, so there are no signs of overconfidence or underconfidence in the models.


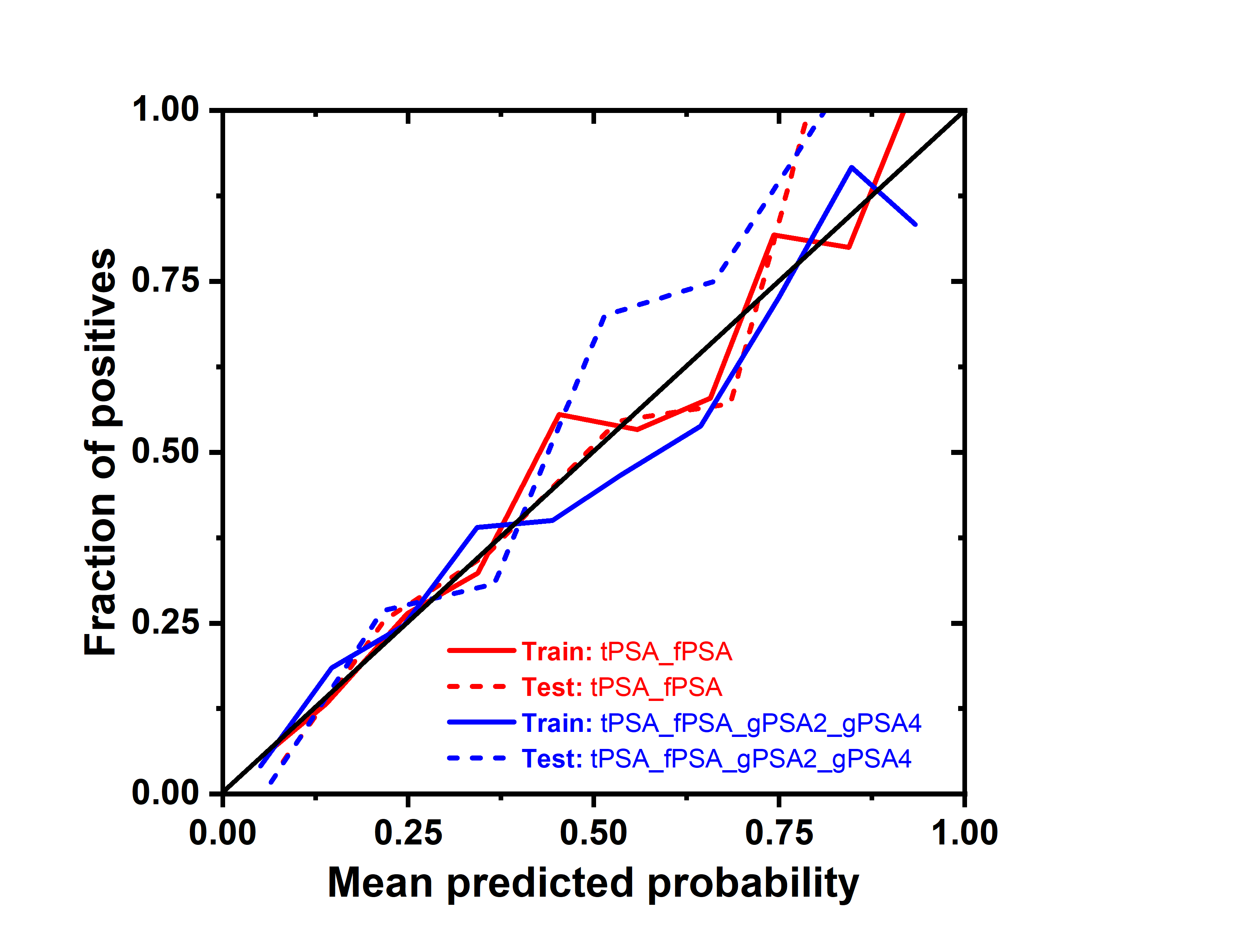


**S3 Figure:** Calibration curve for fPSA_tPSA and GIA for trains and test subsets.

**Glycoprofiling of ZA2G**

Five different ZA2G antibodies (Abcam, UK) were for their use in a ZAG2G glycosylation sandwich ELISA test. ZA2G was immobilised onto the CM5 SPR chip (29-1496-04) and antibodies were injected at six different concentrations (0; 3.7; 11.1; 33.3; 100; 300 nM) in order to identify kinetic and affinity constants and to find the best binding molecule. The SPR experiments (obtained using a Biacore X100 system with all the reagents used in these experiments purchased from GE Healthcare) revealed that a rabbit polyclonal anti-ZA2G (ab47116) exhibited significant SPR binding with maximal SPR response R_max_ of 1758.7 RU with apparent K_D_ in the low nM range. The other antibodies tested, like rabbit polyclonal anti-ZA2G (ab117275), rabbit polyclonal anti-ZA2G (ab133405), rabbit monoclonal anti-ZA2G (ab250225) and rabbit monoclonal anti-ZA2G (ab250135) bound poorly or not at all to ZA2G under the experimental conditions.

PHA-E and WFL lectins did not bind at all to commercial available ZA2G. This is because this ZA2G protein does not contain cancer-related glycans that can be identified by PHA-E and WFL lectins. As mentioned above, this was the case for the fPSA standard as well, confirming that there are no proper commercially available glycoprotein standards for calibrating of the GIA assay.

**References**

1. Sjoberg D. dcurves: Decision Curve Analysis for Model Evaluation. *R package version 03 0.* 2022;537.
